# The Simultaneous Deletion of pH-Sensing Receptors GPR4 and OGR1 (GPR68) Ameliorates Colitis with Additive Effects on Multiple Parameters of Inflammation

**DOI:** 10.3390/ijms26041552

**Published:** 2025-02-12

**Authors:** Federica Foti, Cordelia Schuler, Pedro A. Ruiz, Leonie Perren, Ermanno Malagola, Cheryl de Vallière, Klaus Seuwen, Martin Hausmann, Gerhard Rogler

**Affiliations:** Department of Gastroenterology and Hepatology, University Hospital Zurich, 8091 Zurich, Switzerlandleonie.perren@usz.ch (L.P.); ermanno.malagola@usz.ch (E.M.); gerhard.rogler@usz.ch (G.R.)

**Keywords:** inflammatory bowel disease, pH-sensing G protein-coupled receptors, GPR4, OGR1

## Abstract

G protein-coupled receptors (GPRs), including pro-inflammatory GPR4 and ovarian cancer GPR1 (OGR1/GPR68), are involved in the pH sensing of the extracellular space and have been implicated in inflammatory bowel disease (IBD). Previous data show that a loss of GPR4 or OGR1 independently is associated with reduced intestinal inflammation in mouse models of experimental colitis. In the present manuscript, we investigated the impact of the simultaneous loss of GPR4 and OGR1 in animal models of IBD. To study the effects of combined loss of *Gpr4 Ogr1* in IBD we used the well-established acute dextran sodium sulfate (DSS) and spontaneous *Il10*^−/−^ murine colitis models. Disease severity was assessed using multiple clinical scores (e.g., body weight loss, disease activity score, murine endoscopic index of colitis severity (MEICS) and histological analyses). Real-time quantitative polymerase chain reaction (qPCR), Western blot, and flow cytometry were used to investigate changes in pro-inflammatory cytokines expression and immune cells infiltration. We found that a combined loss of GPR4 and OGR1 significantly reduces colon inflammation in IBD relative to single deficiencies as evidenced by reduced body weight loss, disease score, CD4/CD8 ratio, and *Il1β*, *Il6*, and *Tnf* in the colon. Similarly, in the *II10* deficiency model, the inflammation was significantly ameliorated upon the simultaneous deletion of GPR4 and OGR1, evidenced by a reduction in the MEICS score, colon length, *Tnf* and *Il1β* measurements, and a decrease in the number of macrophages in the colon, as compared to single deletions. Importantly, hydroxyproline levels were decreased close to baseline in *Il10*^−/−^ × *Gpr4*^−/−^ × *Ogr1*^−/−^ mice. Our findings demonstrate that the simultaneous loss of GRP4 and OGR1 functions exerts an additive effect on multiple parameters associated with colonic inflammation. These results further reinforce the hypothesis that chronic inflammatory acidosis is a driver of fibrosis and is dependent on GPR4 and OGR1 signaling. The inhibition of both GPR4 and OGR1 by pH-sensing receptor modulators may constitute as a potential therapeutic option for IBD, as both pH-sensing receptors appear to sustain inflammation by acting on complementary pro-inflammatory pathways.

## 1. Introduction

The maintenance of adequate pH levels, which enable the functioning of internal organs, body fluids, and the body surface, is a basic requirement for the survival of an organism. The pH levels in the body are not uniform but are tailored to the function of each organ, e.g., an acidic environment in the stomach (pH 1–2), on the skin (pH 5.5), or a near-neutral environment in the blood (pH 7.35–7.45), gallbladder (pH 7.1), and saliva (pH 7.1–7.0). Not surprisingly, mammalian organisms have proteins that can respond to changes in pH. Proton-sensing G protein-coupled receptors (GPRs) respond to changes in pH in the extracellular space. Both receptors have a comparable pH range for activation and inactivation, with maximal activation at approximately pH 6.8, and inactivation at a pH greater than 7.8 [1]. GPR4 is highly specific to endothelial cells (ECs) and plays an important role in regulating their functions [2,3,4]. It has been found in colonic arteries, arterioles, capillaries, venules, venular cells [5], and pericytes surrounding vessels. Conversely, ovarian cancer G protein-coupled receptor 1 (OGR1, GPR68) is expressed in various cell types, including highly mobile leukocytes such as monocytes/macrophages, T cells, granulocytes, and various mesenchymal cell lineages that still retain some degree of mobility [6].

Both receptors sense extracellular protons through histidine residues located in the extracellular region, thereby undergoing a conformational change which leads to an activated state. Specifically, GPR4 signals through Gαs [1], Gα_12/13_, and Gα_q11_ [7], which facilitate signaling through cAMP, RhoA, and IP3, respectively. On the other hand, OGR1 signals predominantly through Gα_12/13_ [8,9,10,11] and Gα_q11_ [1]. A specific pH value is essential for the maintenance of organ function, but a changing pH can also serve as a valuable signal for an immunological response. For example, low pH is a hallmark of inflammation in all tissues and organs. Regarding the gut, local acidity is associated with the presence of inflammation and appears to play a role in the initiation and progression of inflammatory processes [12,13]. In chronic inflammation, the local proton concentration is elevated, due mainly to tissue hypoxia, reduced perfusion, and high levels of glycolytic metabolite synthesis [12,13,14,15], leading to pro-inflammatory cytokine production [15]. Importantly, the acidic tissue microenvironment is not only a consequence of inflammation but can actively influence its degree and outcome [16,17,18].

In 2017, there were 6.8 million cases of the chronic form of intestinal inflammation—inflammatory bowel disease (IBD)—globally [19]. IBD is a long-lasting inflammation of the intestine that causes severe damage to the mucus layer. IBD consists of two primary phenotypes: ulcerative colitis (UC) and Crohn’s disease (CD). Several risk factors have been associated with IBD, including genetic susceptibility [20], intestinal microbiota alterations [21], environmental factors [22], and immunological abnormalities [23].

IBD patients have increased GPR4 expression compared to healthy controls [24], implying inflammation-driven angiogenesis. The deletion of GPR4 in mouse models of experimental colitis has been associated with decreased mucosal leukocyte infiltration and reduced intestinal inflammation [24,25]. In a similar manner, intestinal tissue of IBD patients shows increased expression of OGR1 in inflamed tissue [6]. The expression of OGR1 correlates with the clinical scores of IBD patients, indicating that OGR1 has a clinically relevant pro-inflammatory effect [6,26]. The loss of Ogr1 in mice protects from colonic inflammation [26] and fibrosis in the spontaneous colitis mouse model [6]. Moreover, the inhibition of OGR1 ameliorates disease conditions in mice with acute and chronic DSS colitis [27].

In the present manuscript, we investigated the effect of combinatorial loss of GPR4 and OGR1 in animal models of IBD. Double KO animals were used in the mouse models of acute DSS-induced colitis and immune-mediated spontaneous inflammation driven by interleukin (*Il*)-*10* deficiency. We were able to show that the simultaneous loss of both receptors ameliorates colonic inflammation in an additive manner with respect to several relevant parameters, demonstrating non-overlapping biological pathways controlled by GPR4 and OGR1, respectively.

## 2. Results

### 2.1. Simultaneous Loss of GPR4 and OGR1 Reduces Inflammation in DSS-Induced Acute Colitis with Additive Effects on Multiple Parameters

To investigate the interplay between GPR4 and OGR1 in the development of acute intestinal inflammation we used the well-characterized DSS model. Double (*Gpr4*^−/−^ × *Ogr1*^−/−^) and single (*Gpr4*^−/−^, *Ogr1*^−/−^) knockout animals were analyzed alongside WT controls.

**Figure 1:**
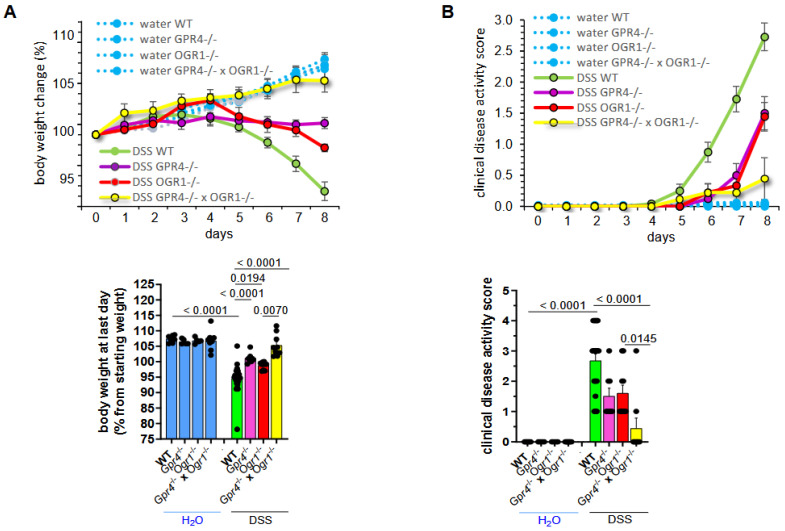

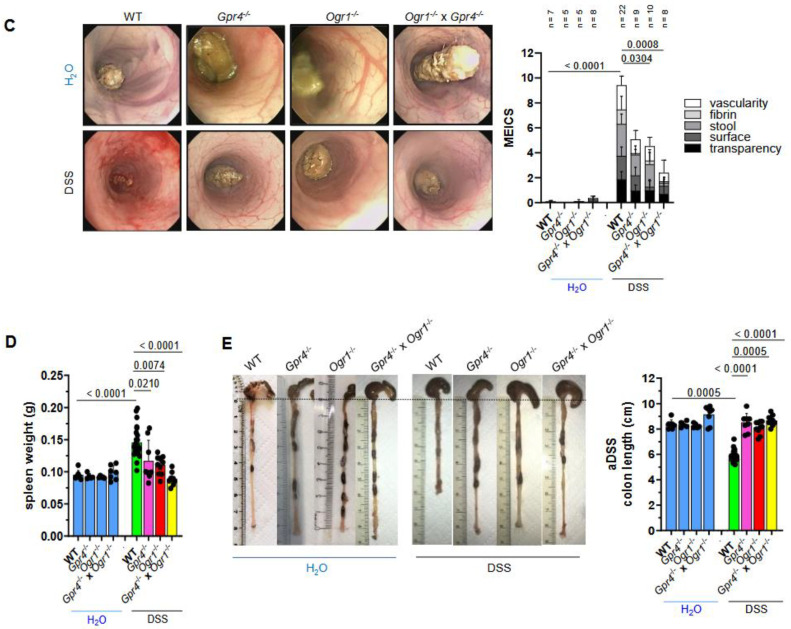
The absence of both GPR4 and OGR1 additively reduces inflammation in DSS-induced colitis. (**A**) Body weight, ±SEM. (**B**) Clinical disease activity score, ±SEM. (**C**) MEICS, ±SD, and exemplary pictures of colonoscopy from each group. (**D**) Spleen weight, ±SD. (**E**) Colon length, ±SD, and exemplary pictures of colons from each group. Non-parametric distribution (Shapiro–Wilk test). One-way ANOVA, multiple comparisons test, Kruskal–Wallis test, two-stage linear step-up procedure of Benjamini, Krieger, and Yekutieli. *p*-values and *n* as indicated.

Briefly, 12–15-week-old WT (*n* = 22), *Gpr4*^−/−^ (*n* = 9), *Ogr1*^−/−^ (*n* = 14), and *Gpr4*^−/−^ × *Ogr1*^−/−^ × (*n* = 4) littermate mice were exposed to DSS for 8 days, resulting in pronounced colitis. As a control, WT (*n* = 7), *Gpr4*^−/−^ (*n* = 5), *Ogr1*^−/−^ (*n* = 5), and *Gpr4*^−/−^ × *Ogr1*^−/−^ (*n* = 8) mice received water (H_2_O) for the same time period. Notably, control groups showed no differences in body weight irrespective of their genotype (Figure 1A). As previously observed, DSS-treated *Gpr4*- and *Ogr1*-deficient mice showed significantly less body weight loss (Figure 1A) compared with the corresponding littermate WT controls (*p* < 0.0001 and *p* = 0.0194, respectively). The body weights of the double KO mice were significantly increased when compared with *Ogr1*^−/−^ mice (*p* = 0.0070) upon DSS (Figure 1A), suggesting an additive effect in the double KO. The disease activity score was significantly decreased in double KO mice compared with the corresponding WT (*p* < 0.0001) and *Ogr1*^−/−^ (*p* = 0.0145) mice, respectively (Figure 1B).

Endoscopy analysis at the time of euthanasia indicated a significant decrease in the murine endoscopic index of colitis severity (MEICS) scores in *Ogr1*-deficient mice and double KO mice relative to WT mice (*p* = 0.0304, *p* = 0.0008, respectively, Figure 1C).

**Figure 2:**
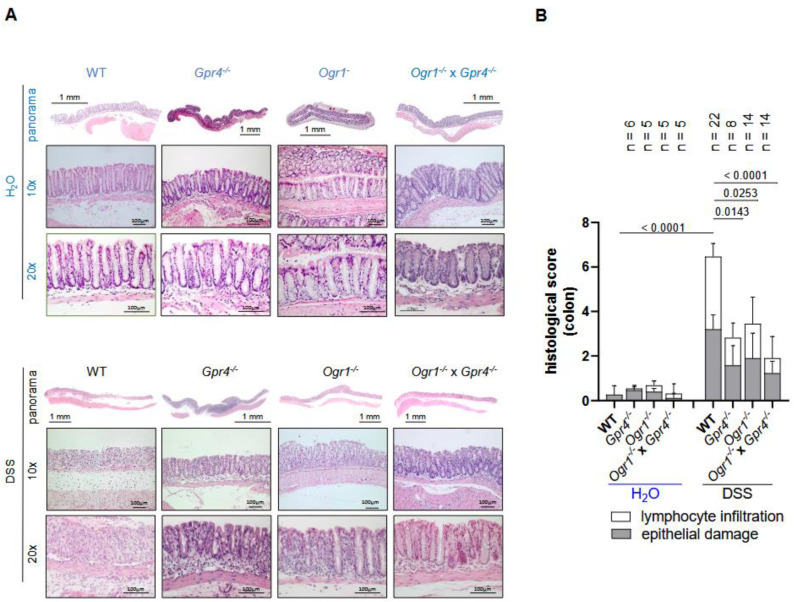
Decreased histological score in *Gpr4*^−/−^ × *Ogr1*^−/−^ compared with WT mice upon acute DSS-induced colitis. (**A**) Exemplary microscopic pictures of HE-stained colons. (**B**) Histological score, non-parametric distribution (Shapiro–Wilk test). One-way ANOVA, multiple comparisons test, Kruskal–Wallis test, two-stage linear step-up procedure of Benjamini, Krieger, and Yekutieli. ±SD, *p*-values, and *n* as indicated.

Regarding inflammation, spleen weight measured post-mortem was significantly decreased in *Gpr4*^−/−^, *Ogr1*^−/−^, and double KO compared with WT mice upon DSS, indicating reduced leukocyte accumulation (*p* = 0.0210, *p* = 0.0074, *p* < 0.0001, respectively, Figure 1D). *Gpr4*^−/−^, *Ogr1*^−/−^, and double KO mice exhibited significantly greater colon length when compared with WT mice upon DSS (*p* < 0.0001, *p* = 0.0005, *p* < 0.0001, respectively, Figure 1E). Moreover, we performed histological analyses to evaluate the severity of inflammation in colon specimens from all groups using HE staining (Figure 2A). Lymphocyte infiltration and epithelial damage were used to calculate the histologic score. The histologic score of *Gpr4*-, *Ogr1*-deficient, and double KO H_2_O control mice showed no significant differences when compared with that of the WT H_2_O control mice (Figure 2B). The histologic score was significantly lower in *Gpr4*^−/−^, *Ogr1*^−/−^, and *Gpr4*^−/−^ × *Ogr1*^−/−^ mice compared with WT mice with DSS-induced acute colitis (*p* = 0.0052, *p* = 0.0118, and *p* < 0.0001, respectively)

### 2.2. Loss of Both Gpr4 and Ogr1 Decreases F4/80^+^ and CD4/CD8 Ratio During Acute Colitis

**Figure 3:**
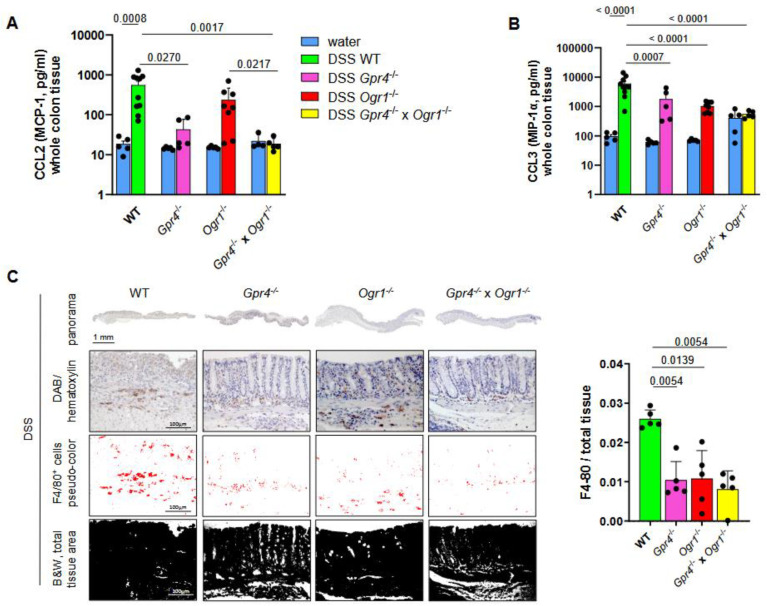

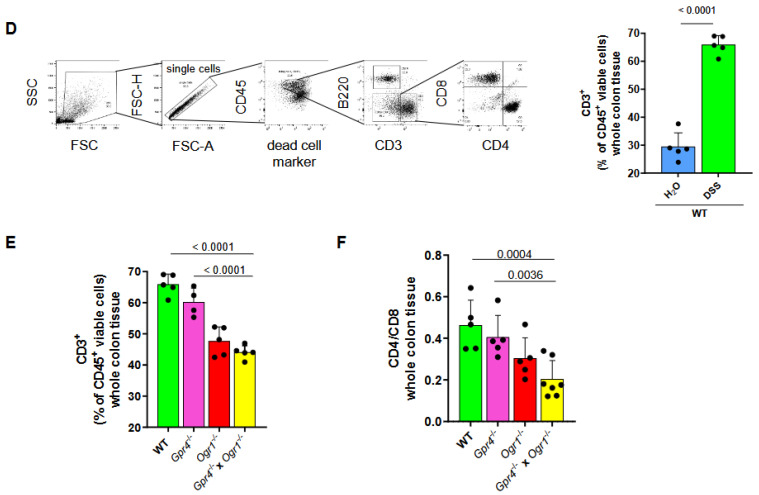
Decreased F4/80^+^ and CD4/CD8 in *Gpr4*^−/−^ × *Ogr1*^−/−^ compared with WT mice upon acute DSS-induced colitis. Immunoassay, (**A**) MCP-1, and (**B**) CCL3 in whole colon tissue, ±SEM each. (**C**) IHC, F4/80, ±SD. Flow cytometry for (**D**,**E**) CD3, ±SD, and (F) CD4/CD8, ±SD. (D) Unpaired *t*-test. (**A**,**C**) Non-parametric distribution (Shapiro–Wilk test). (**B**,**D**–**F**) Normal distribution (Shapiro–Wilk test). (**A**–**C**,**E**,**F**) One-way ANOVA, multiple comparisons test, Kruskal–Wallis test, two-stage linear step-up procedure of Benjamini, Krieger, and Yekutieli. *p*-values and *n* as indicated.

To investigate the effect of simultaneous loss of GPR4 and OGR1 in acute intestinal inflammation we analyzed cytokine levels via multiplex analysis. Whole colon tissue from WT (*n* = 10), *Gpr4*^−/−^ (*n* = 5), *Ogr1*^−/−^ (*n* = 8), and *Gpr4*^−/−^ × *Ogr1*^−/−^ (*n* = 5) littermate mice exposed to DSS for 8 days, and control WT (*n* = 5), *Gpr4*^−/−^ (*n* = 5), *Ogr1*^−/−^ (*n* = 5), and *Gpr4*^−/−^ × *Ogr1*^−/−^ (*n* = 5) mice exposed to water (H_2_O) for the same duration were evaluated. No differences in the expression of MCP-1 (CCL2) were observed in H2O-treated mice irrespective of their genotype (Figure 3A and Figure S1). CCL2 was significantly decreased in *Gpr4*^−/−^ mice compared with the corresponding WT mice (*p* = 0.0270) with DSS-induced acute colitis. The *Gpr4*^−/−^ × *Ogr1*^−/−^ mice upon DSS showed decreased CCL2 levels compared with the WT and *Ogr1*^−/−^ mice upon DSS (*p* = 0.0017 and *p* = 0.0217, respectively). MIP-1α (CCL3) was significantly decreased in *Ogr1*^−/−^, *Gpr4*^−/−^, and double KO mice compared with DSS-induced acute colitis with the corresponding WT mice (*p* = 0.0007, *p* < 0.0001, *p* < 0.0001, respectively, Figure 3B). IHC was used to assess the presence of macrophages expressing the cell marker F4/80 in whole colon tissue (Figure 3C). Consistent with the decrease in macrophage attractant protein CCL2, and CCL3, which is mainly produced by macrophages, the number of F4/80-positive cells was significantly increased in *Gpr4*^−/−^, *Ogr1*^−/−^, and double KO mice compared to WT mice (*p* = 0.0054 *p* = 0.0139 *p* = 0.0054, respectively, *n* = 5 each) with DSS-induced acute colitis.

We determined the changes in the number of T cells in whole colon tissue by flow cytometry. The results showed a significant increase in CD3^+^ cells in WT mice with DSS-induced acute colitis compared to WT mice exposed to H_2_O (*p* < 0.0001, *n* = 5 each), indicating the successful initiation of intestinal inflammation (Figure 3D, based on markers for viable, CD45^+^, B220^−^, CD3^+^, Table S1). The proportion of CD3^+^ in the double KO mice was significantly decreased when compared to both WT and *Gpr4*^−/−^ mice upon DSS (*p* < 0.0001, both, Figure 3E). In addition, the CD4/CD8 ratio was significantly decreased when compared with WT and *Gpr4*^−/−^ mice (*p* = 0.0004 and *p* = 0.0036, respectively, Figure 3F), suggesting an additive effect in the double KO. The decrease in the number of macrophages, CD3^+^ cells, and CD4/CD8 ratio in double KO mice in whole colon tissue reflects protection against inflammatory processes.

**Figure 4:**
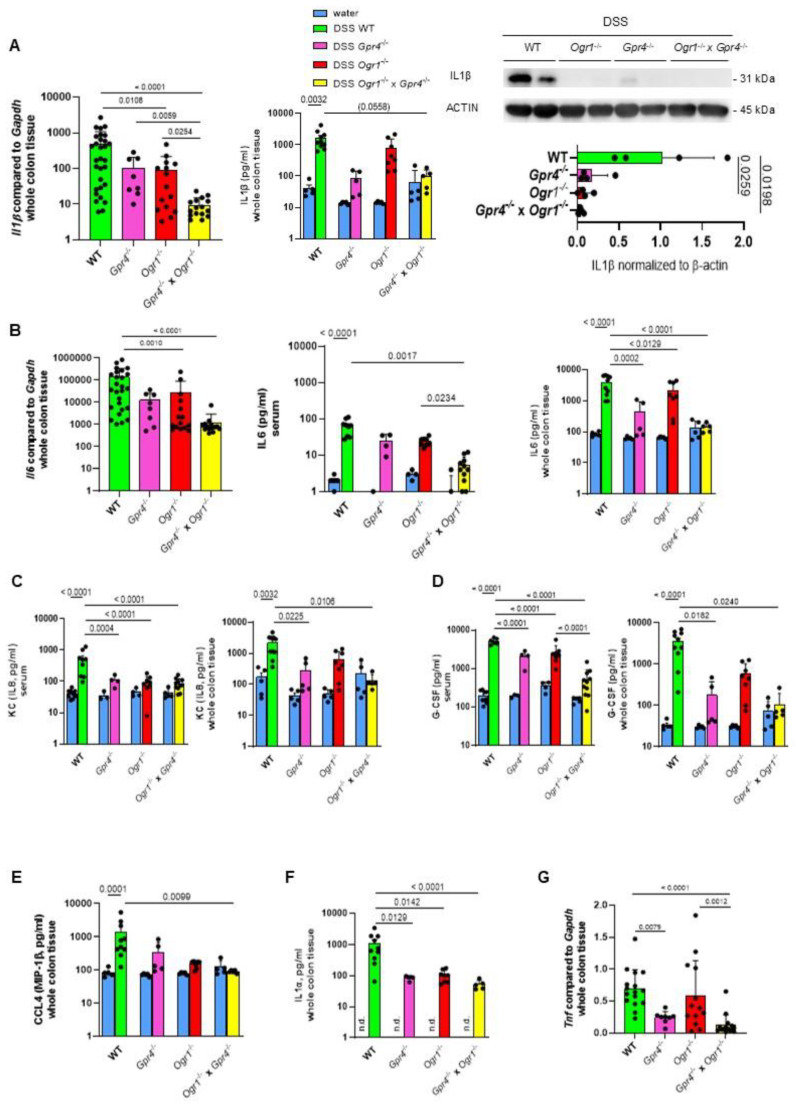
GPR4 and OGR1 deficiency additively reduces pro-inflammatory cytokines in acute DSS-induced colitis. (**A**) *Il1β.* qPCR, ±SD; immunoassay, ±SEM; and WB, ±SD, in whole colon tissue each. (**B**) *Il6*. qPCR, ±SD and immunoassay, ±SEM, in whole colon tissue and serum as indicated. (**C**) KC, (**D**) G-CSF, (**E**) CCL4, and (**F**) IL1α in serum or whole colon tissue as indicated, ±SEM. (**G**) qPCR, *Tnf*, ±SD. (**A**–**G**) One-way ANOVA, multiple comparisons test, Kruskal–Wallis test, two-stage linear step-up procedure of Benjamini, Krieger, and Yekutieli. *p*-values and *n* as indicated. Non-parametric distribution, except IL-6 whole colon tissue, KC serum, and G-CSF serum, which showed normal distribution (Shapiro–Wilk test).

Whole colon tissue was analyzed for the presence of mRNA for the pro-inflammatory cytokine *Il1β* (Figure 4A). *Ogr1*^−/−^ mice showed significantly decreased *Il1β* expression compared to WT upon DSS (*p* = 0.0108), which was further decreased in double KO mice compared to *Ogr1*^−/−^ mice (*p* = 0.0254), indicating an additive effect in the double KO and at the same time an ameliorated course of inflammation. Multiplex analysis showed a trend toward decreased IL1β protein in the double KO. Western blot analysis confirmed the significant decrease in IL1β in both *Ogr1*^−/−^ mice and double KO mice compared to WT mice (*p* = 0.0259 and *p* = 0.0198, respectively).

Notably, expression levels of pro-inflammatory cytokine *Il6* were significantly reduced in the colon tissue of double KO mice. In line with these results, serum levels of IL6 were also reduced. Both serum and whole colon tissue were analyzed for the presence of mRNA for the (Figure 4B). *Ogr1*^−/−^ mice and double knockout mice showed significantly decreased *Il6* mRNA expression compared to WT upon DSS (*p* = 0.0010, *p* < 0.0001, respectively). Multiplex analysis of serum showed significantly decreased IL6 in the double KO mice compared to WT mice (*p* = 0.0017). Significantly decreased IL6 in the double KO mice compared to *Ogr1*^−/−^ mice indicates an additive effect (*p* = 0.0234) and ameliorated inflammation. In whole colon tissue, IL6 was significantly decreased in *Ogr1*^−/−^, *Gpr4*^−/−^, and double KO mice compared with DSS-induced acute colitis with the corresponding WT mice (*p* = 0.0002, *p* = 0.0129, *p* < 0.0001, respectively).

Both serum and whole colon tissue were analyzed for the presence of KC (Figure 4C) and G-CSF (Figure 4D) by multiplex analysis. KC showed significantly decreased protein levels in double KO mice compared to WT mice (*p* < 0.0001 and *p* = 0.0106, respectively, Figure 4C). *Gpr4*^−/−^, *Ogr1*^−/−^, and double knockout mice showed significantly decreased G-CSF expression in serum samples compared to WT upon DSS (*p* = 0.0001 each, Figure 4D). The significantly decreased G-CSF in the double KO mice compared to *Ogr1*^−/−^ mice indicates an additive effect (*p* < 0.0001).

Whole colon tissue was analyzed for the presence of CCL4 (MIP-1β, Figure 4E) and IL1α (Figure 4F) by multiplex analysis. Both proteins showed significantly decreased protein levels in double KO mice compared to WT mice (*p* < 0.0001 each). Whole colon tissue was analyzed for the presence of mRNA for the pro-inflammatory cytokine *Tnf* (Figure 4G). Double KO mice showed significantly decreased *Tnf* mRNA expression compared to WT and *Ogr1*^−/−^ mice upon DSS (*p* < 0.0001, *p* = 0.0012, respectively), indicating ameliorated inflammation.

### 2.3. The Absence of Both GPR4 and OGR1 Reduces Inflammation in Spontaneous Colitis

**Figure 5:**
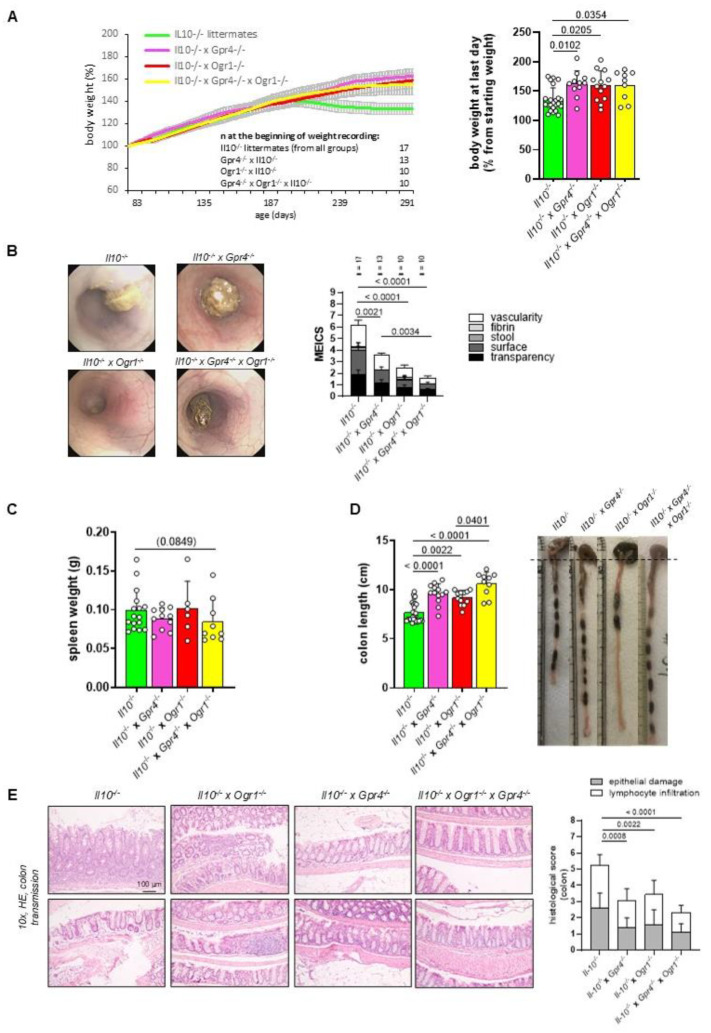
The absence of both GPR4 and OGR1 reduces inflammation upon spontaneous colitis. (**A**) Body weight, ±SEM. (**B**) MEICS, ±SD. (**C**) Spleen weight. (**D**) Colon length, ±SD, and exemplary pictures of colons from each group. (**E**) Exemplary microscopic pictures of HE-stained colons and histological score. Non-parametric distribution (Shapiro–Wilk test). One-way ANOVA, multiple comparisons test, Kruskal–Wallis test, two-stage linear step-up procedure of Benjamini, Krieger, and Yekutieli. *p*-values and n as indicated.

To confirm the interaction of GPR4 and OGR1 in the development of intestinal inflammation we applied the spontaneous colitis model which is driven by IL10 deficiency. *Il10*^−/−^ (*n* = 17), *Il10*^−/−^ × *Gpr4*^−/−^ (*n* = 13), *Il10*^−/−^ × *Ogr1*^−/−^ (*n* = 10), and *Il10*^−/−^ × *Gpr4*^−/−^ × *Ogr1*^−/−^ (*n* = 10) developed colitis after approximately 190 days. *Il10*^−/−^ × *Gpr4*^−/−^, *Il10*^−/−^ × *Ogr1*^−/−^, and *Il10*^−/−^ × *Gpr4*^−/−^ × *Ogr1*^−/−^ mice showed significantly less body weight loss on the day of the euthanasia (291 days) compared with the corresponding *Il10*^−/−^ mice (Figure 5A, *p* = 0.0102, *p* = 0.0205, and *p* = 0.0354, respectively). Endoscopy was performed on all mice prior to euthanasia (Figure 5B). The resulting MEICS score showed significantly decreased scores for *Il10*^−/−^ × *Gpr4*^−/−^, *Il10*^−/−^ × *Ogr1*^−/−^, and *Il10*^−/−^ × *Gpr4*^−/−^ × *Ogr1*^−/−^ compared with *Il10*^−/−^ mice (*p* = 0.0021, *p* < 0.0001, and *p* < 0.0001, respectively). The MEICS score of the *Il10*^−/−^ × *Gpr4*^−/−^ × *Ogr1*^−/−^ mice was significantly decreased when compared with *Il10*^−/−^ × *Gpr4*^−/−^ mice (*p* = 0.0034), suggesting an additive effect. Spleen weight measured post-mortem showed a trend toward a decrease in *Il10*^−/−^ × *Gpr4*^−/−^ × *Ogr1*^−/−^ mice compared with WT mice (*p* = 0.0849, Figure 5C). *Il10*^−/−^ × *Gpr4*^−/−^, *Il10*^−/−^ × *Ogr1*^−/−^, and *Il10*^−/−^ × *Gpr4*^−/−^ × *Ogr1*^−/−^ mice exhibited significantly greater colon length when compared with the corresponding *Il10*^−/−^ mice (*p* < 0.0001, *p* = 0.0022, and *p* < 0.0001, respectively, Figure 5D). The significantly greater colon length in *Il10*^−/−^ × *Gpr4*^−/−^ × *Ogr1*^−/−^ mice compared to *Il10*^−/−^ × *Ogr1*^−/−^ mice indicates an additive effect (*p* = 0.0401).

Histological samples were used to evaluate lymphocyte infiltration and epithelial damage to calculate the histological score in colon samples using HE staining (Figure 5E). The histologic score was significantly lower in *Il10*^−/−^ × *Gpr4*^−/−^, *Il10*^−/−^ × *Ogr1*^−/−^, and *Il10*^−/−^ × *Gpr4*^−/−^ × *Ogr1*^−/−^ mice when compared with the corresponding *Il10*^−/−^ mice (*p* = 0.0008, *p* = 0.0022, and *p* < 0.0001, respectively, Figure 5E).

### 2.4. The Absence of Both GPR4 and OGR1 Reduces CD4/CD8 Ratio and Macrophages in Spontaneous Colitis

**Figure 6:**
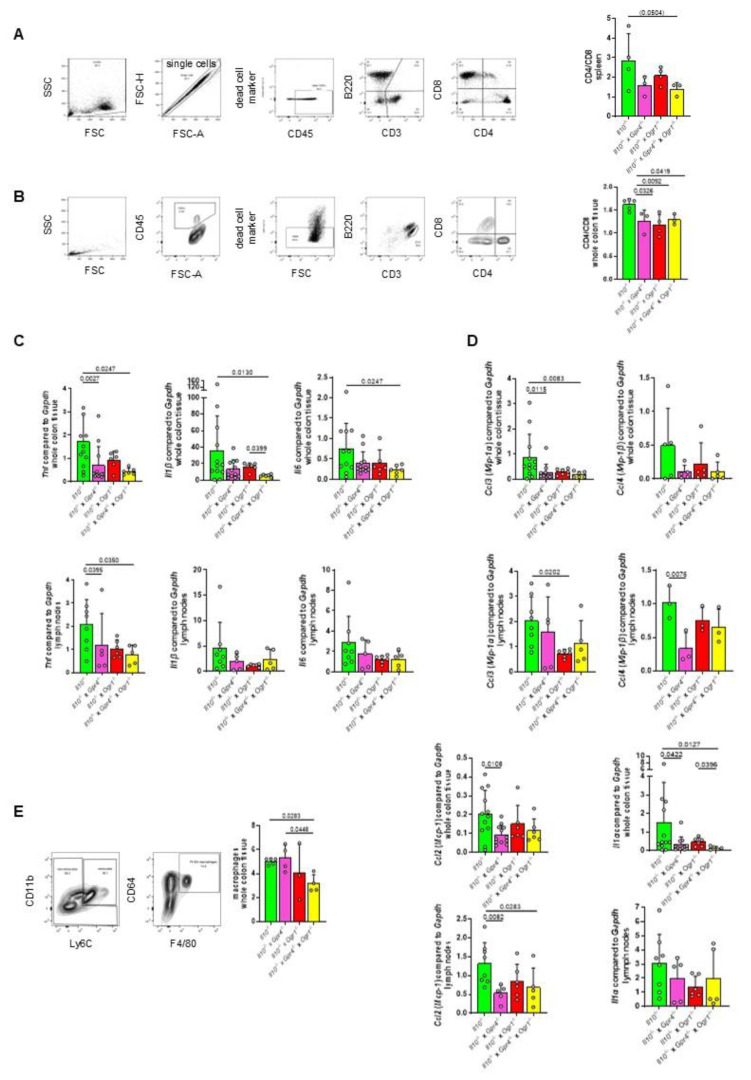
The absence of both GPR4 and OGR1 reduces CD4/CD8 ratio and macrophages upon spontaneous colitis. Flow cytometry for CD4/CD8 in (**A**) spleen, and (**B**) whole colon tissue. (**C**) qPCR, pro-inflammatory *Tnf*, *Il1β*, and *Il6* in whole colon tissue and lymph nodes as indicated. (**D**) qPCR, monocyte-attracting *Ccl3*, and *Ccl4*. (**E**) Flow cytometry for F4/80^+^ in whole colon tissue and qPCR, *Ccl2*, and *Il1α* mainly produced by monocytes. Non-parametric distribution (Shapiro–Wilk test) except for (**B**), (**D**) lymph nodes, (**E**) *Ccl2* lymph nodes. ±SD, one-way ANOVA, multiple comparisons test, Kruskal–Wallis test, two-stage linear step-up procedure of Benjamini, Krieger, and Yekutieli. *p*-values and *n* as indicated.

To analyze the CD4/CD8 ratio in the development of spontaneous intestinal inflammation, we determined the changes in the spleen and the whole colon tissue by flow cytometry. In the spleen, the CD4/CD8 ratio showed a trend toward a decrease in *Il10*^−/−^ × *Gpr4*^−/−^ × *Ogr1*^−/−^ mice when compared with the corresponding *Il10*^−/−^ mice (*p* = 0.0504, Figure 6A, based on markers for viable, CD45^+^, B220^−^, CD3^+^, CD4^+^, CD8^+^ cells, Table S2). In whole colon tissue, the CD4/CD8 ratio was significantly lower in *Il10*^−/−^ × *Gpr4*^−/−^, *Il10*^−/−^ × *Ogr1*^−/−^, and *Il10*^−/−^ × *Gpr4*^−/−^ × *Ogr1*^−/−^ mice when compared with the corresponding *Il10*^−/−^ mice (*p* = 0.0326, *p* = 0.0092, and *p* = 0.0419, respectively, Figure 6B).

Both whole colon tissue and lymph nodes were analyzed for the presence of mRNA for the pro-inflammatory cytokines *Tnf*, *Il1β*, and *Il6* (Figure 6C). qPCR analysis of whole colon tissue showed significantly decreased mRNA in *Il10*^−/−^ × *Gpr4*^−/−^ × *Ogr1*^−/−^ mice when compared with the corresponding *Il10*^−/−^ mice (*p* = 0.0247, *p* = 0.0103, and *p* = 0.0247, respectively). qPCR analysis of lymph nodes showed significantly decreased *Tnf* mRNA in *Il10*^−/−^ × *Gpr4*^−/−^ × *Ogr1*^−/−^ mice when compared with the corresponding *Il10*^−/−^ mice (*p* = 0.0350) and a trend towards a decrease for *Il1β* and *Il6*.

Both whole colon tissue and lymph nodes were analyzed for the presence of monocyte-attracting *Ccl3* and *Ccl4* mRNA (Figure 6D). qPCR analysis of whole colon tissue showed significantly decreased *Ccl3* mRNA in *Il10*^−/−^ × *Gpr4*^−/−^ × *Ogr1*^−/−^ mice when compared with the corresponding *Il10*^−/−^ mice (*p* = 0.0083) and a trend towards a decrease in lymph nodes. *Ccl4* qPCR analysis showed a trend towards a decrease in *Il10*^−/−^ × *Gpr4*^−/−^ × *Ogr1*^−/−^ mice when compared with the corresponding *Il10*^−/−^ mice for both whole colon tissue and lymph nodes. To assess the recruitment of macrophages to the whole colon tissue, we determined the changes in the number of F4/80^+^ macrophages by flow cytometry. Flow cytometry showed a significant increase in F4/80^+^ cells in *Il10*^−/−^ × *Gpr4*^−/−^ × *Ogr1*^−/−^ mice when compared with the corresponding *Il10*^−/−^ mice, indicating ameliorated intestinal inflammation (*p* = 0.0283, Figure 6E, based on markers for viable, CD45^+^, B220^−^, CD3^−^, negative for CD11b/Ly6C, CD64^+^, F4/80^+^, Table S3). The significantly decreased number of F4/80^+^ cells in *Il10*^−/−^ × *Gpr4*^−/−^ × *Ogr1*^−/−^ mice compared to *Il10*^−/−^ × *Gpr4*^−/−^ mice indicates an additive effect (*p* = 0.0448). In addition, both whole colon tissue and lymph nodes were analyzed for the changes in mRNA for *Ccl2* and *Il1α*, which are mainly produced by monocytes (Figure 6E). qPCR analysis of whole colon tissue showed a trend toward a decrease in *Ccl2* mRNA in *Il10*^−/−^ × *Gpr4*^−/−^ × *Ogr1*^−/−^ mice when compared with the corresponding *Il10*^−/−^ mice, and a significant decrease in lymph nodes (*p* = 0.0283). qPCR analysis of whole colon tissue showed a significant decrease for *Il1α* mRNA in *Il10*^−/−^ × *Gpr4*^−/−^ × *Ogr1*^−/−^ mice when compared with the corresponding *Il10*^−/−^ mice (*p* = 0.0127). The significant decrease in Il1α mRNA in *Il10*^−/−^ × *Gpr4*^−/−^ × *Ogr1*^−/−^ mice compared to *Il10*^−/−^ × *Ogr1*^−/−^ mice indicates an additive effect (*p* = 0.0396).

### 2.5. The Absence of Gpr4 and Ogr1 Reduces Fibrosis in Spontaneous Colitis

**Figure 7:**
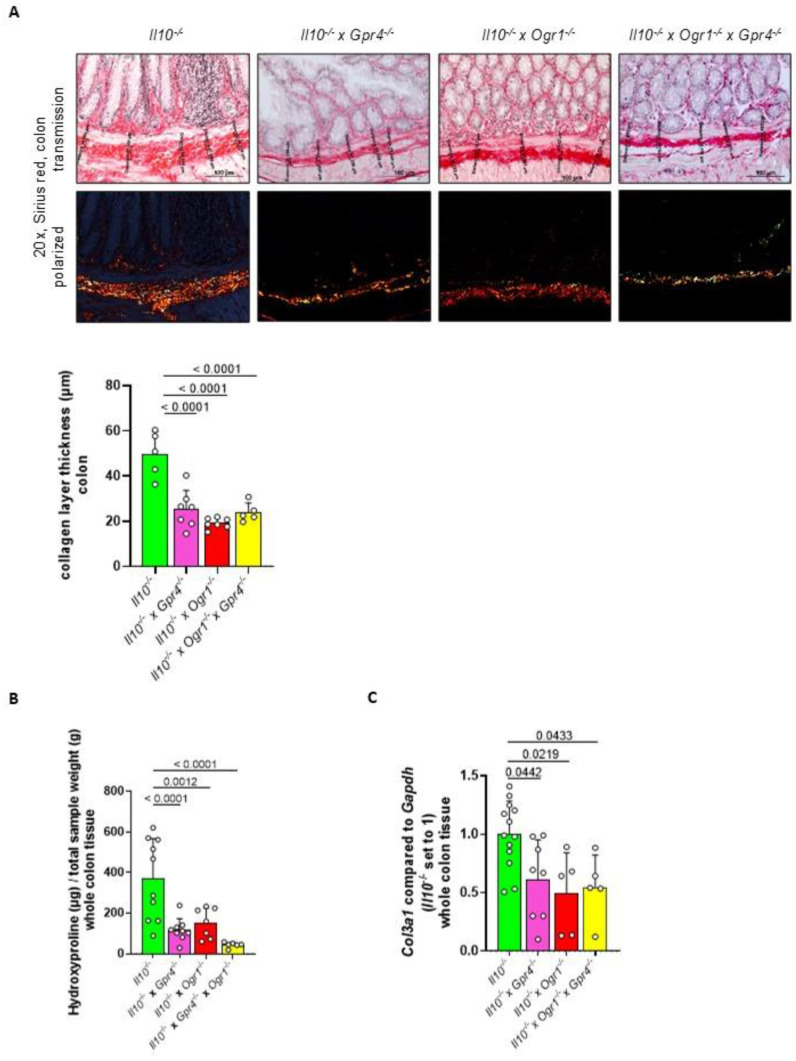
Decreased fibrosis in *Gpr4*^−/−^ × *Ogr1*^−/−^ compared with WT mice upon spontaneous colitis. (**A**) Sirius red staining and collagen layer thickness. (**B**) Hydroxyproline assay. (**C**) qPCR, *Col3a1*. Normal distribution (Shapiro–Wilk test), one-way ANOVA, multiple comparisons test, Kruskal–Wallis test, two-stage linear step-up procedure of Benjamini, Krieger, and Yekutieli, ±SD, *p*-values, and *n* as indicated.

To analyze the severity of fibrosis in the development of spontaneous intestinal inflammation, we determined the changes in the whole colon tissue by Sirius red staining, hydroxyproline assay, and *Col3a1* qPCR. Histological samples were used to evaluate fibrosis in colon specimens from all groups using Sirius red staining to highlight collagen (Figure 7A). Collagen layer thickness was significantly reduced in *Il10*^−/−^ × *Gpr4*^−/−^, *Il10*^−/−^ × *Ogr1*^−/−^, and *Il10*^−/−^ × *Gpr4*^−/−^ × *Ogr1*^−/−^ mice when compared with the corresponding *Il10*^−/−^ mice (*p* < 0.0001, each). To confirm the changes in collagen deposition in whole colon tissue, we performed a hydroxyproline assay. Hydroxyproline content was significantly reduced in *Il10*^−/−^ × *Gpr4*^−/−^, *Il10*^−/−^ × *Ogr1*^−/−^, and *Il10*^−/−^ × *Gpr4*^−/−^ × *Ogr1*^−/−^ mice when compared with the corresponding *Il10*^−/−^ mice (*p* < 0.0001, *p* = 0.0012, and *p* < 0.0001, respectively, Figure 7B and Figure S2). In addition, whole colon tissue was analyzed for the changes in mRNA for *Col3a1*, which is mainly produced by fibroblasts during fibrogenesis (Figure 7C). qPCR analysis of whole colon tissue showed a significant decrease for *Col3a1* mRNA in *Il10*^−/−^ × *Gpr4*^−/−^, *Il10*^−/−^ × *Ogr1*^−/−^, and *Il10*^−/−^ × *Gpr4*^−/−^ × *Ogr1*^−/−^ mice compared to *Il10*^−/−^ mice. In summary, changes in whole colon tissue determined by Sirius red staining, hydroxyproline assay, and *Col3a1* qPCR reflect a decreased collagen deposition and ameliorated fibrogenesis in *Il10*^−/−^ × *Gpr4*^−/−^ × *Ogr1*^−/−^ mice.

## 3. Discussion

In the present study, we investigated the role of the pro-inflammatory proton-sensing receptors GPR4 and OGR1 using well-established murine models of acute and spontaneous colitis. GPR4 is found on ECs [1,2,3], OGR1 is found on vascular pericytes [10], smooth muscle [28], and highly motile leukocytes [29]. We found that the simultaneous loss of both receptors ameliorates colonic inflammation in an additive manner with respect to several relevant parameters, demonstrating non-overlapping biological pathways controlled by GPR4 and OGR1, respectively.

In the model of DSS-induced acute colitis, anti-inflammatory effects were particularly pronounced in double KO animals in terms of reduced body weight loss, disease score, CD4/CD8 ratio, and *Il1β*, *Il6*, and *Tnf* expression in the colon. In the *Il10* deficiency model, the anti-inflammatory effects, and the anti-inflammatory effects in the double KO animals were particularly pronounced in the MEICS score, colon length, *Tnf* and *Il1β* measurements, and the decreased number of macrophages in the colon. Of note, hydroxyproline levels were decreased in *Il10*^−/−^ × *Gpr4*^−/−^ × *Ogr1*^−/−^ mice to almost baseline levels, suggesting that chronic inflammatory acidosis, sensed by GPR4 and OGR1, is critically required to drive fibrosis.

Antagonists of GPR4 and OGR1 may offer a therapeutic opportunity for IBD, and the pharmaceutical industry has already started to develop modulators of pH-sensing receptors. Allosteric modulators of the proton-sensing receptor GPR4, such as the imidazopyridine derivative 39c, which was developed as an orally active inhibitor of GPR4 for the treatment of inflammatory diseases, show potent cellular activity and are effective in animal models of inflammation [4]. Importantly, 39c also reduced collagen deposition in a murine model of gut fibrosis [30]. These data further corroborate results from *Gpr4*-deficient mice in models of intestinal fibrosis, indicating that collagen deposition is reduced in the absence of GPR4 signaling. A close analog of derivative 39c, the GPR4 inhibitor compound 13 (also known as NE-52-QQ57), reduced clinical severity and macroscopic disease indicators of intestinal inflammation in a mouse model of acute colitis [31]. Histidines are proposed to represent orthosteric sites [32], but they are arranged differently on GPR4 and OGR1 [33]. Therefore, it may be unlikely to find a high-potency pan-antagonist. Indeed, other imidazopyridine derivatives designed as allosteric modulators of the proton-sensing GPR4 inhibited responses mediated by GPR4 but not those mediated by OGR1 [34]. A small molecule OGR1 inhibitor (OGR1-I) was tested in a mouse model of acute DSS-induced colitis [27] and was shown to reduce clinical severity and ameliorate inflammation. In addition, OGR1-I reduced T-cell infiltration in acute colitis and macrophage recruitment in chronic colitis. The inhibition of GPR4 and OGR1 activity by pharmacological intervention may represent a promising novel approach to reduce inflammation by attenuating leukocyte infiltration into inflamed tissues. It will be interesting to further define in greater detail the interplay of these complementary pathways in IBD.

This study has limitations. The DSS-induced colitis model involves tissue damage and mainly reflects the immunological situation during acute inflammation, while *Il10*-deficient mice are useful in determining the long-term effects of ongoing inflammation initiated spontaneously. In comparison, defining features of IBD include severe and long-lasting mucosal tissue destruction interrupted by gradually shorter remission phases. Further studies are needed to study the effects of the double KO in an animal model that recapitulates the relapsing–remitting pattern of IBD.

## 4. Materials and Methods

### 4.1. Animals

All animal experiments were performed according to the ARRIVE criteria and approved by the Veterinary Authority of the Canton of Zurich (registration number ZH 113/2021). The generation, breeding, and genotyping of B6.Balb/c-GPR4tm1Lud (*Gpr4*^−/−^, then bred onto a C57BL/6 background) have been described previously [2]. C57BL/6 B6-Gpr68<tm1Dgen> (*Ogr1*^−/−^) were obtained from Deltagen, Inc., San Mateo, CA, USA. [35]. Double KO mice (*Gpr4*^−/−^ × *Ogr1*^−/−^) were bred from the above mice and genotyped. Littermates were used in all experiments. The animals were co-housed wherever possible, and bedding was exchanged among the cages to minimize the potential effects of microbiota variation. All the animals were housed in a specific pathogen-free facility. The animals were kept in type II long clear-transparent individually ventilated cages (IVCs, 365 mm × 207 mm × 140 mm, Allentown, N.J., USA) with autoclaved dust-free bedding and tissue papers as nesting material. They were fed a pelleted and extruded mouse diet (R/M–H Extrudat, ssniff Spezialdiäten, Soest, Germany) ad libitum. The light/dark cycle in the room was given through natural daylight (sunrise: 07:00 h, sunset: 18:00 h). The mice were weighed at 10:00 h every morning. The temperature was set to 21  ±  1 °C, with a relative humidity of 55  ±  5% and 75 complete changes of filtered air per hour (filter: Megalam MD H14, Camfil, Zug, Switzerland).

### 4.2. DSS-Induced Acute Colitis

*Gpr4*^−/−^, *Ogr1*^−/−^, and *Gpr4*^−/−^ × *Ogr1*^−/−^ mice were each mated separately in a heterozygous manner. The resulting homozygous receptor-deficient offspring and wildtypes were used for the experiments. All wildtypes of the three groups were considered as single experimental groups. This results in a different group size for the wildtypes used. Acute colitis was induced by the addition of 2.5% DSS (36–50 kDa, MP Biomedicals, California, CA, USA) in the drinking water for 8 consecutive days. Mouse body weight and clinical phenotype were assessed daily.

### 4.3. Spontaneous Il10 Deficient Colitis Model

B6.129P2-Il10<tm1Cgn>/J (*Il10*^−/−^) mice were crossed with the above-mentioned strains to generate mice susceptible to spontaneous colitis (*Gpr4*^−/−^ × *Il10*^−/−^, *Ogr1*^−/−^ × *Il10*^−/−^, and *Gpr4*^−/−^ × *Ogr1*^−/−^ × *Il10*^−/−^). Groups of genetically modified mice were mated separately in a heterozygous manner for pH-sensing receptors, but a homozygous manner for Il10. Thus, all resulting offspring was *Il10*^−/−^. All *Il10*^−/−^ animals of the three groups were considered as single experimental groups. This results in a different group size for the *Il10*^−/−^ used. Mice were observed for 291 days and mouse body weight and clinical phenotype were assessed.

### 4.4. Clinical Disease Activity Score

Clinical scoring of the animals was based on a clinical score established to monitor severity in an experimental colitis model. For each animal, clinical parameters including stool consistency and general clinical condition were assessed and scored to quantify the disease severity on a daily basis (Table 1). Body weight loss was determined separately.

### 4.5. Assessment of Colonoscopy

Prior to endoscopic assessment, the animals were anesthetized intraperitoneally with a mixture of 90–120 mg ketamine (Narketan 10%, Vétoquinol AG, Bern Switzerland) and 8 mg xylazine (Rompun 2%, Bayer, Switzerland) per kg body weight and examined with the Tele Pack Pal 20,043,020 (Karl Storz Endoskope, Tuttlingen, Germany). Mice were scored with a murine endoscopic index of colitis severity (MEICS) score (Table 2) [36].

### 4.6. Serum and Tissue Collection for Histology and Molecular Analysis

At the end of DSS-induced acute colitis or spontaneous colitis, mice were euthanized, and the entire gastrointestinal tract was removed. The length of the colon was measured from the anus to the ileocecal junction and then dissected from the cecum.

One centimeter of the distal third of the colon was removed for histologic analysis. Five-millimeter sections of the colon were resected commencing from the anus and moving toward the cecum and immediately snap frozen in liquid nitrogen for storage at a −80 °C freezer for RNA analysis, Western blot, multiplex, hydroxyproline assay. Spleens were removed for post-mortem spleen weight measurement and splenocyte isolation for flow cytometry.

Serum was collected from mice after DSS for multiplex assay. Inguinal lymph nodes were isolated from Il10-deficient mice. The lymph nodes were immediately snap-frozen in liquid nitrogen and stored at −80 °C in a freezer for RNA analysis.

### 4.7. Histological Score in Mice

For the assessment of the histological scores, 1 cm of the distal third of the colon was removed, fixed in 10% buffered formalin, embedded, stained with hematoxylin and eosin (HE), and scored as described [37,38]. Briefly, mice were scored individually. Histology was performed by an independent investigator blinded to the type of treatment. Histology was scored as follows:

Epithelium (E) 0: normal morphology; 1: loss of goblet cells; 2: loss of goblet cells in large areas; 3: loss of crypts; 4: loss of crypts in large areas.

Infiltration (I) 0: no infiltrate; 1: infiltrate around the crypt base; 2: infiltrate extending to the *L. muscularis mucosae*; 3: extensive infiltrate extending to the *L. muscularis mucosae* and mucosal thickening with abundant edema; 4: infiltration of the *L. submucosa*.

The total histologic score is the sum of the epithelial and infiltration scores and ranges from 0 to 8 (total score = E + I).

### 4.8. Flow Cytometry

For flow cytometry analysis, lamina propria lymphocytes and splenocytes were isolated. Surface antigens were stained with a mix of antibodies including a viability marker (Tables S1–S3) and incubated at 4 °C for 20 min. After washing with phosphate-buffered solution (PBS) and centrifugation, all the samples were fixed. For fixation, BD Cytofix/Cytoperm (554722, Becton Dickinson, Franklin Lakes, NJ, USA) and BD Perm/Wash (554723, Becton Dickinson, Franklin Lakes, NJ, USA ) were used following the manufacturer’s instructions. The pellet was then resuspended in PBS. Data were acquired on a flow cytometer LSR II Fortessa 4L (Becton Dickinson, Franklin Lakes, NJ, USA) and analyzed with the FlowJo software (version 10.2, Becton Dickinson, Franklin Lakes, NJ, USA).

### 4.9. Lamina Propria Lymphocytes Isolation

Hanks’ balanced salt solution (HBSS) was prepared from the solid salts (Sigma, Hanks’ Balanced Salts, H2387–10X1L, Sigma-Aldrich, Burlington, MA, USA) according to the manufacturer’s instructions. The enzyme mixture for tissue digestion was prepared as follows: 0.4 mg/mL collagenase type IV (Gibco, 17104-019, Gibco, New York, NY, USA) and 0.6 mg/mL dispase (Gibco, 17105-041, Gibco, New York, NY, USA) were added to 10% fetal calf serum (FCS) low Ig (PAN Biotech, P30-2802, Wimborne, UK) in HBSS colorless (Sigma, H8264-1L, Burlington, MA, USA). Cells were isolated from 2 cm of the distal colon per sample. The samples were first transferred in 2 mM EDTA (Sigma Aldrich, 38057-1EA, Burlington, MA, USA) in HBSS for 15 min at 37 °C while shaking. The supernatant was removed, and the samples were incubated in 2 mM EDTA in HBSS for another 30 min. The supernatant was removed again, and the samples were incubated in warm HBSS before transfer to the previously prepared enzyme mix for tissue digestion and incubated for 20 min at 37 °C with shaking. After incubation, the samples were sheared with the help of a syringe and 18 G needle and then passed through a 70 µm cell strainer. After centrifugation, the supernatant was removed, and the cells were resuspended in PBS before staining and fixation.

### 4.10. Splenocyte Isolation

Spleen samples were minced, passed through a 70 µm cell strainer with PBS, and centrifuged. The resulting pellet was then lysed with 3 mL ACK buffer (NH_4_Cl 0.15 M, KHCO_3_ 0.01 M, EDTA-Na_2_ 0.001 M) for 3 min at room temperature. After resuspension with PBS, the solution was filtered through a 70 µm cell strainer and centrifuged again. The pellet was then resuspended in PBS before the cells were stained and fixed.

### 4.11. Compensation Controls

Two different bead kits were used for compensation controls: ArC Amine reactive compensation bead kit (Thermo Fisher Scientific, A10346, Waltham, MA, USA) for the cell viability marker. For the remaining antibodies, the BD CompBeads kit anti-Rat and anti-Hamster (Becton Dickinson Pharmingen Biosciences, 552845, Franklin Lakes, NJ, USA), and the BD CompBeads kit anti-Mouse (Becton Dickinson Pharmingen Biosciences, 552843, Franklin Lakes, NJ, USA) were used. All the compensation controls were prepared following the manufacturer’s instructions.

### 4.12. Immunohistochemistry (IHC) and Densitometry

Specimens were fixed in 4% PBS-buffered formalin, embedded in paraffin, sectioned (3 µm), and deparaffinized. H&E staining was performed according to standard procedures. Antigen retrieval of tissues for IHC was performed in citrate buffer, pH 6.0 (Agilent DAKO, Santa Clara, CA, USA) at 98 °C for 30 min. Inhibition of endogenous peroxidases was performed by incubating tissue slides in 0.9% hydrogen peroxide for 15 min at RT. Serum to block non-specific binding was used for 1 h at RT. F4/80 expressed by macrophages was stained with monoclonal rat anti-mouse antibody (T-2006, 1:50, BMA Biomedicals AG, Augst, Switzerland). Primary antibodies were diluted in blocking solutions and slides were incubated overnight at 4 °C. For IHC samples, a secondary HRP-conjugated antibody (ImmPRESS, Vector Laboratories, Newark, CA, USA) was applied for 1 h at RT, and staining was visualized using the 3,3′-diaminobenzidine (DAB) ImmPACT Peroxidase Substrate Brown Kit (Vector Laboratories, Newark, CA, USA). IHC specimens were counterstained with hematoxylin, dehydrated, and mounted.

Staining was examined using the Imager Z2 microscope and the software ZEN. Briefly, 20x magnification pictures from at least three representative areas from each section were taken using transmission light microscopy. The relative quantity of the according antigen was analyzed using the Fiji software (1.52a, NIH). The total tissue area was determined by converting the image into an 8-bit image type and adjusting the B&W threshold. The area covered with DAB was determined by setting thresholds to select the brown color. The quantity of the according antigen was calculated as the area of brown color/total tissue area.

### 4.13. Sirius Red Staining and Collagen Layer Thickness

After deparaffinization, Sirius Red staining was performed to highlight collagen (red staining) according to a standard protocol [39]. Collagen layer thickness was determined by an independent investigator blinded to the type of experiment. Microscopic evaluation was performed using an AxioCam MRc5 (Zeiss, Jena, Germany) on a Zeiss Axiophot microscope (Zeiss, Jena, Germany). Collagen layer thickness was measured using the AxioVision Release 4.7.2 software (Zeiss; menu items: measure, length). Thickness was calculated from at least eight locations i representative areas (in at least five samples examined for each genotype). Layer thickness data were normally distributed and calculated using the Kruskal–Wallis test, a Benjamini, Krieger, and Yekutieli two-stage linear step-up procedure.

### 4.14. Hydroxyproline Determination

Hydroxyproline content was quantitated from freshly isolated small bowel and grafts using the chloramine T colorimetric method according to the manufacturer’s protocol (MAK008-1KT, Sigma-Aldrich, Burlington, MA, USA). Tissues (20–50 mg) were weighed, homogenized using gentleMACS Octo Dissociator (130-096-427Miltenyi Biotec, Bergisch Gladbach, Germany), and hydrolyzed in 500 µL of 6 N HCl at 100 °C for 24 h. Samples (20–50 µL) were dried at 90 °C. Dried samples were incubated each with 50 μL chloramine T/oxidation buffer mixture (3 μL chloramine T concentrate and 47 μL oxidation buffer) at room temperature for 5 min; 50 μL diluted DMAB reagent (25 μL dimethylaminobenzaldehyde, 25 μL perchloric acid/isopropanol) was subsequently added to each sample, and incubation was carried out at 60 °C for 90 min for chromophore formation. Absorbance was read at 560 nm.

### 4.15. Multiplex Cytokines Analysis

Cytokine concentration in cell culture supernatants was measured using a Bio-Plex Pro Mouse Cytokine assay (#M60009RDPD, Bio-Rad Laboratory, Hercules, CA, USA) according to the manufacturer’s instructions. The assay was used to detect monocyte chemoattractant protein-1 (MCP-1/CCL2), macrophage inflammatory protein-1α (MIP-1α/CCL3), interleukin (IL)1β, IL6, keratinocyte-derived chemokine (KC, a functional IL8-homolouge in mouse), IL1α, chemokine (C-C motif) ligands 4 (CCL4, previously known as macrophage inflammatory protein, MIP-1β), and granulocyte colony-stimulating factor (G-CSF). Data were collected and analyzed using a Bio-Rad BioPlex 200 instrument equipped with Bio-Plex Manager software version 6.0 (Bio-Rad Laboratory, Hercules, CA, USA).

### 4.16. Ribonucleic Acid (RNA) Isolation, Complementary DNA (cDNA) Synthesis, and qPCR

Total RNA was isolated from the colon and ileum using the Maxwell RSC simplyRNA tissue kit (Promega, Madison, WI, USA, AS1340). For all samples, lysis buffer from the kit was added to snap frozen resections, and samples were shredded in M tubes (Miltenyi Biotec, Bergisch Gladbach, Germany) using a gentleMACS tissue homogenizer (Miltenyi Biotec, Bergisch Gladbach, Germany). RNA concentration was determined by absorbance at 260 and 280 nm with a NanoDrop (Thermo Fisher Scientific, Waltham, MA, USA). cDNA synthesis was performed using a High-Capacity cDNA Reverse Transcription Kit (Applied Biosystems, Foster City, CA, USA) following the manufacturer’s instructions. qPCR was performed using the TaqMan Fast Universal Master Mix (Applied Biosystems, Foster City, CA, USA) on a QuantStudio™ 6 Flex Real-Time PCR System and the results were analyzed with the SDS software (Applied Biosystems, Foster City, CA, USA). For each sample, triplicates were measured, and glyceraldehyde-3-phosphate dehydrogenase (*Gapdh*) was used as endogenous control. Results were analyzed using the ∆∆CT method. The following gene expression assays for mouse were used: *Col3a1* Mm01254476_m1, *Il1β* Mm00443259_g1, *Il6* Mm00446190_m1, tumor necrosis factor (*Tnf)* Mm00443259_g1, and *Gapdh* 4352339E (Thermo Fisher Scientific, Waltham, MA, USA).

### 4.17. Western Blot (WB)

All the samples were snap-frozen in liquid nitrogen prior to storage at -80 °C. For protein isolation, the samples were homogenized together with 400 µL isolation buffer: 1 tablet cOmplete Mini (Roche, 11836170001) in 10 mL M-PER (78501, Thermo Fisher Scientific, Waltham, MA, USA) lysis buffer, using the gentleMACS Octo dissociator (130-095-937, Miltenyi Biotec, Bergisch Gladbach, Germany). After processing and centrifugation, the supernatant was collected. Protein concentration was measured by NanoDrop (protein A280 program, double determination, Thermo Fisher Scientific, Waltham, MA, USA) according to the manufacturer’s instructions.

A total of 20 µg of protein dissolved in 17.5 µL was combined with 6.25 µL NuPage LDS Sample Buffer (NP0007, Thermo Fisher Scientific, Waltham, MA, USA) and 1.25 µL DTT 1M (R0861). Protein was size separated on 10% sodium dodecyl sulfate acrylamide gel with Tris-glycine-sodium dodecyl sulfate running buffer running at 200 V according to standard protocol. Protein ladder Precision Plus Dual Color standards (1610374, Bio-Rad, Hercules, CA, USA) and SeeBlue™ Plus2 (LC5925, Thermo Fisher Scientific, Waltham, MA, USA) were used as size references.

The proteins were then transferred to a nitrocellulose membrane (0.2 µm pore; Thermo Fisher Scientific, Waltham, MA, USA). For blotting, the voltage was set at 30 V. The membrane was blocked for 1 h at room temperature in WB wash buffer (2% 0.5 M tris pH 6.8, 2% 5 M NaCl, 0.1% Tween 20 from Sigma in double-deionized water) containing 3% bovine serum albumin (BSA, P06-1391500, PAN Biotech, Wimborne, UK) and 3% milk powder (T145.3, Carl Roth, Arlesheim, Switzerland).

Primary antibodies: Goat polyclonal anti-mouse IL-1β (R&D AF-401-NA, 1:1000), and mouse monoclonal anti-pan-actin-antibody (MAB1501, 1:10000, EMD Millipore, Burlington, MA, USA) in 3% BSA in WB wash buffer. Secondary antibody: Horseradish peroxidase–conjugated donkey anti-goat IgG-HRP (sc-2020, 1:5000, Santa Cruz Biotechnology Inc., Santa Cruz, CA, USA) and goat anti-mouse IgG-HRP (sc-2005, 1:10000, Santa Cruz Biotechnology Inc., Santa Cruz, CA, USA). Immunodetection was performed with a WesternBright ECL kit (K-12045-D209, Advansta Inc., San Jose, CA, USA), according to the manufacturer’s instructions on Fusion Solo S (Vilber Lourmat, Collégien, France) using the EvolutionCapt edge software (Vilber Lourmat, Collégien, France). Densitometry was performed using Fiji software (version 2.1.0).

### 4.18. Statistical Analysis

Statistical analysis was performed using GraphPad Prism (v 9.4.1). The parametric distribution was calculated using the Shapiro–Wilk test. Differences were calculated using a T-test, one-way ANOVA, a multiple comparisons test, a Kruskal–Wallis test, or a two-stage linear step-up procedure of Benjamini, Krieger, and Yekutieli, as indicated in the figure legends. Differences were considered significant at a *p*-value < 0.05. Results are presented as mean ± standard deviation (SD) or ± standard error of the mean (SEM), as indicated in the figure legends.

## Figures and Tables

**Table 1:** Clinical disease activity score parameters.

Appearance	0 1 2 3 4	normal (normal well-groomed, smooth fur, clean orifices of the body, clear eyes) marginal (slightly disordered fur, eyes not clotted) minor (dull fur, untended, some orifices of the body marginally clotted) moderate (rough coat, some orifices of the body clotted) elevated (orifices clearly clotted, abnormal body posture)
behavior/activity	0 1 2 3 4	normal (social interaction, curiosity, grouped sleeping with others, reacts to touch) marginal deviation (reduced movement when cage is opened, reduced interest) mild (less active, but still in the group) moderate (increased inactivity, keeps isolated) elevated (almost no movement)
Pain	0 1 2 3	no (relaxed body and moving behavior) minor (active, but seldom belly tension observed, no hunching, normal eyes) mild (occasionally wide gait, belly tension more frequent, hunching) severe (frequent belly tension, hunched back, breathing frequency elevated, body temperature reduced, squinted eyes)
Stool consistency	0 1 2 3 4	solid soft, but formed pellets soft, no pellets formed very soft, but still with solid particles liquid
Blood in stool	0 1 2	no blood in stool sporadically visible (but dry) traces of blood in stool blood in stool or in cage clearly detectable fresh blood in cage clearly visible or rectal blood loss clearly visible
Weight loss	0 1 2 3 4 5	stable or weight gain <5% 5–10% 11–15% 16–20% ≥20%

**Table 2:** MEICS score parameters.

**Thickening of the colon**	0 1 2 3	transparent moderate marked non-transparent
**Changes in the vascular pattern**	0 1 2 3	normal moderate marked bleeding
**Fibrin visible**	0 1 2 3	none little marked extreme
**Granularity of the mucosal surface**	0 1 2 3	none moderate marked extreme
**Stool consistency**	0 1 2 3	normal and solid still shaped unshaped spread

## Data Availability

The data underlying this article are available in a repository provided by the University of Zurich.

## Supplementary Materials

The following supporting information can be downloaded at: https://www.mdpi.com/article/10.3390/ijms26041552/s1.

## Abbreviations

cAMP	cyclic adenosine monophosphate
CCL4	chemokine (C-C motif) ligands 4
CD	Crohn’s disease
cDNA	complementary DNA
DSS	dextran sodium sulfate
EC	endothelial cell
Gapdh	glyceraldehyde-3-phosphate dehydrogenase
G-CSF	granulocyte colony-stimulating factor
FSC	forward scatter
GPRs	G protein-coupled receptors
GPR4	G protein-coupled receptor 4
IBD	inflammatory bowel disease
HBSS	Hank’s balanced salt solution
HE	hematoxylin and eosin
IL	interleukin
KC	keratinocyte-derived chemokine
MCP-1	monocyte chemoattractant protein-1
MEICS	murine endoscopic index of colitis severity
MIP-1α	macrophage inflammatory protein-1α
OGR1	ovarian cancer GPR 1 (GPR68)
PBS	phosphate-buffered solution
qPCR	real-time quantitative polymerase chain reaction
SSC	side scatter
Tnf	tumor necrosis factor
UC	ulcerative colitis
WT	wildtype
