# Supplementary material for: The Simultaneous Deletion of pH-Sensing Receptors GPR4 and OGR1 (GPR68) Ameliorates Colitis with Additive Effects on Multiple Parameters of Inflammation
Source: Int J Mol Sci. 2025 Feb 12;26(4):1552. doi: 10.3390/ijms26041552 (PMC11855581; doi:10.3390/ijms26041552)

International Journal of
Molecular Sciences

MDPI

Article

The Simultaneous Deletion of pH-Sensing Receptors GPR4 and OGR1 (GPR68) Ameliorates Colitis with Additive Effects on Multiple Parameters of Inflammation

Federica Foti [1], Cordelia Schuler [1], Pedro A. Ruiz [1], Leonie Perren [1], Ermanno Malagola [1], Cheryl de Vallière [1], Klaus Seuwen [1], Martin Hausmann [1,*,†] and Gerhard Rogler [1,†]

Supplementary files

Table S1. Antibodies used for single-cell analysis of whole colon tissue in the flow cytometer for the acute DSS-induced colitis model (M = mouse; R = rat; H = hamster; NA = not applicable).

Fluorochrome	Target	Manufacturer	Ref. number	Dilution	Host
PacificBlue	CD45	BioLegend	103126	1:400	R
APC-Cy7	viability marker	BioLegend	423106	1:200	NA
FITC	CD3	BioLegend	100306	1:200	H
BV785	B220	BioLegend	103246	1:200	R
BV650	CD4	BioLegend	100546	1:200	R
PE-Texas Red	CD8a	BD Biosciences	562283	1:400	R

Table S2. Antibodies used for single-cell analysis of splenocytes in the flow cytometer for the spontaneous colitis model (M = mouse; R = rat; H = hamster; NA = not applicable).

Fluorochrome	Target	Manufacturer	Ref. number	Dilution	Host
PacificBlue	CD45	BioLegend	103126	1:400	R
APC-Cy7	viability marker	BioLegend	423106	1:200	NA
PE-Cy5.5	CD3	Invitrogen	35-0031-82	1:200	H
PE-Cy5	B220	Invitrogen	15-0452-83	1:200	R
BV650	CD4	BioLegend	100546	1:200	R
PE-Texas Red	CD8a	BD Biosciences	562283	1:400	R

Table S3. Antibodies used for single-cell analysis of whole colon tissue in the flow cytometer for the spontaneous colitis model (M = mouse; R = rat; H = hamster; NA = not applicable).

Fluorochrome	Target	Manufacturer	Ref. number	Dilution	Host
PacificBlue	CD45	BioLegend	103126	1:400	R
APC-Cy7	viability marker	BioLegend	423106	1:200	NA
PE-Cy5.5	CD3	Invitrogen	35-0031-82	1:200	H
APC	B220	BioLegend	103212	1:200	R
BV650	CD4	BioLegend	100546	1:200	R
PE-Texas Red	CD8a	BD Biosciences	562283	1:400	R
BV711	Ly6C	BioLegend	128037	1:200	R
BV605	CD11b	BioLegend	101257	1:200	R
FITC	F4/80	eBioscience	11-4801-85	1:200	R
DsRed, PE	CD64	BioLegend	139304	1:100	M

Figure S1: Immunoassay, standard curves.

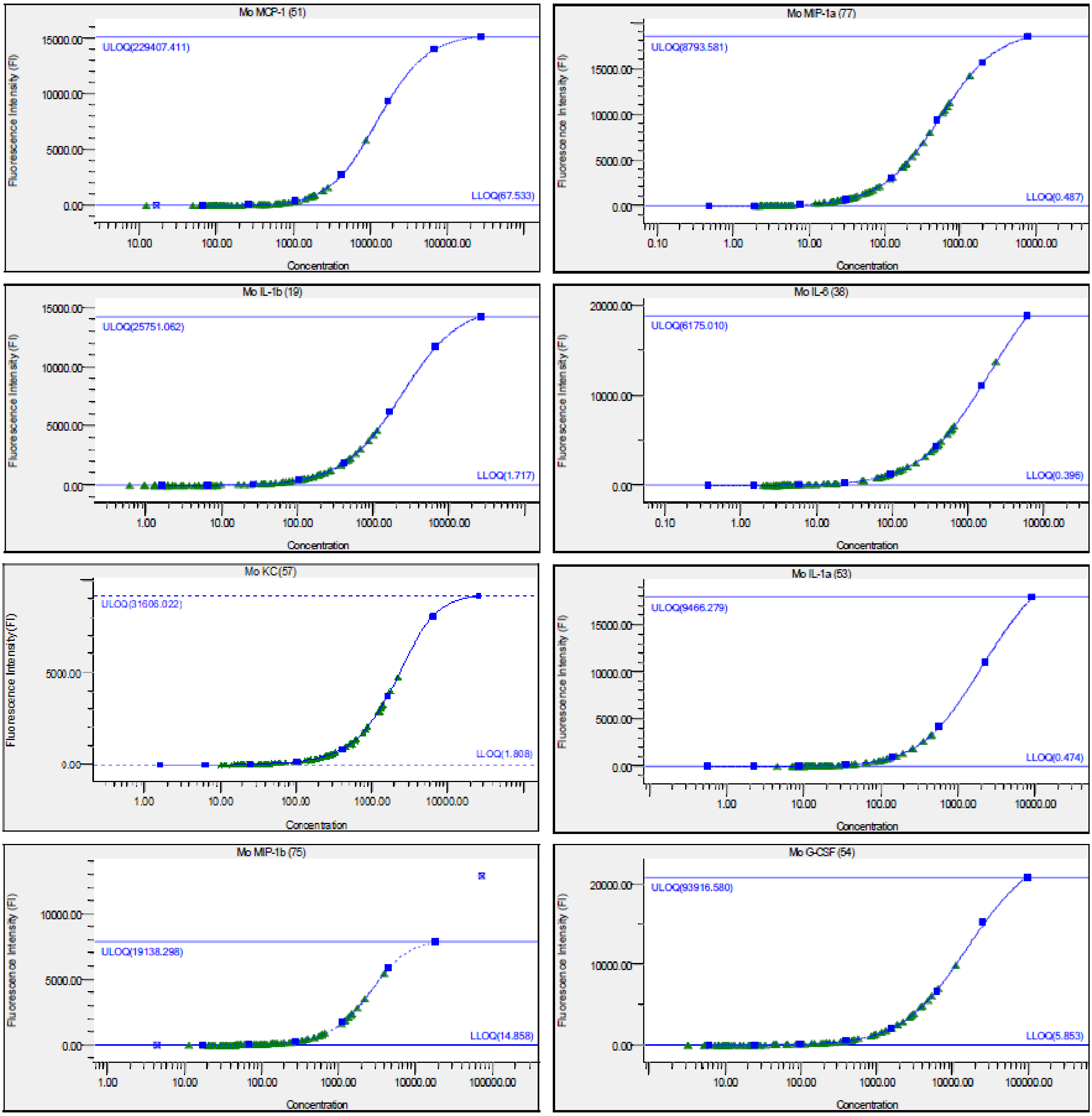

Figure S2. Hydroxyproline assay, standard curve.

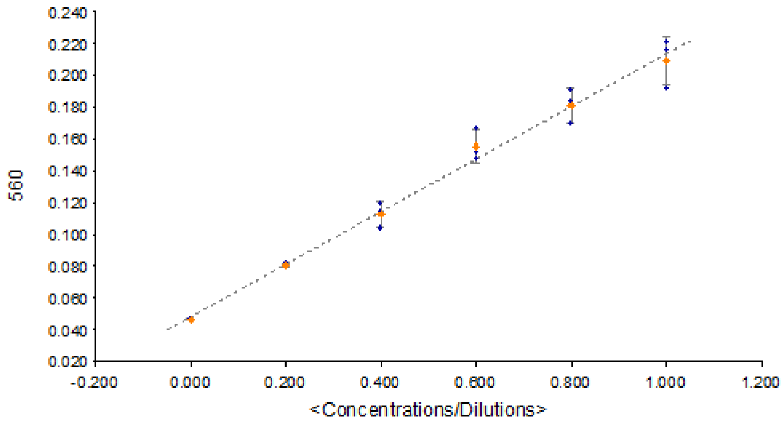

Supplement: Supplementary file 1 [file ijms-26-01552-s001.zip › ijms-3377182-supplementary.pdf]
